# Daytime sleep following observational practice does not enhance a visual-spatial and a motor representation

**DOI:** 10.1007/s00221-026-07281-2

**Published:** 2026-04-02

**Authors:** Stefan Panzer, Nils Hofstetter, Moritz Schreiber, David L. Wright

**Affiliations:** 1 Saarland University, Universität Campus Gebäude B8.2, D-66123 Saarbrücken, Germany; 2 Texas A&M University, College Station, USA

**Keywords:** Sequence learning, Representation, Inter-manual transfer, Consolidation

## Abstract

The present study examined if a daytime nap of 90 min can further improve skill memory afforded by observation and physical practice. Moreover, we determined if a ‘nap’ benefit was restricted to a particular representational form (spatial or motor) that can be used to govern performance. Participants were assigned to one of four experimental conditions: physical practice with no nap, physical practice with a nap, observation with no nap, and observation with a nap. Specifically, after either physical or observational practice of a 1300-ms sequence of flexion and extensions at the right elbow, a participant experienced a 90-min nap or an equivalent period of wake-filled rest. Each participant then completed a retention test and a set of inter-manual transfer tests that probed the efficacy of spatial and motor representations that developed because of the practice modality and if a nap was experienced. As expected, retention and transfer performance were superior following physical practice compared to observation. However, observation offered benefits to skill revealed by lower RMSE for observers at retention compared to that displayed during the initial trials for the individuals’ privy to physical practice. A nap only supported a reduction in error when administered after physical practice. This was true not only for retention but also during tests that relied on available visual-spatial and motor representations. Counter to recent reports, observers did not benefit from a nap. A nap following observation harmed individuals to instantiate the visual-spatial and the motor representation for movement sequence production.

## Introduction

Research during the last two decades has provided empirical evidence that the execution of a movement sequence can be guided by a spatial representation framed in allocentric coordinates and/or a motor representation referencing the egocentric context. Both representations are developed during the time course of practice (e.g. Albouy et al. 2013; Bapi et al. 2006; Shea et al. 2011; Shea and Wright 2012). These findings are consistent with the *Parallel Network Model* which proposed that movement sequence learning occurs simultaneously and independently in visual-spatial and motor forms (Hikosaka et al. 1999). The visual-spatial representation develops relatively quickly, is coded abstractly (i.e., is effector-independent), and is the dominant representation early in practice (Shea et al. 2011). Alternatively, a motor representation develops more slowly, considers an individual’s movement dynamics to optimize the use of the specific effectors being used (i.e., is effector-dependent), and is the dominant representation late in practice (Shea et al. 2011).

Thea assessment of visual-spatial and motor representations of skill is illustrated in Panzer et al. (2009) who required individuals to practice a simple spatial–temporal sequence of elbow extension and flexions in 1300-ms for 99 trials in a single training session. To determine the development of both a visual-spatial and motor representation following training, participants were administered two separate transfer tests after retention was assessed. During the first effector transfer test, the visual-spatial coordinates present for the practice movement sequence were reinstated but the sequence was performed with the unpracticed limb. Thus, for this test, referred to as a *non-mirrored* transfer test, participants moved to the same spatial locations but used a novel pattern of muscle activation and joint angles to successfully execute the novel sequence. In a second transfer test, called the *mirror* transfer test, again using the untrained limb, the participant performed the same pattern of muscle activation and the same relative joint angles as those performed during practice while using the contralateral homologous muscles. For this test, a novel set of spatial locations was traversed. Thus, from exposure to 99 trials of the original movement sequence, superior performance emerged for the mirror rather than the non-mirror transfer test. This finding indicates that this practice extent was adequate to support the development of a motor representation for the initially trained movement sequence, which was sufficient to support better performance than when only the visual spatial reference was used.

While physical practice is frequently the impetus for the development of sequence representations for action, the spatial form can be developed through observation of a novel sequence. For instance, Gruetzmacher et al. (2011) conducted an experiment using the same movement sequence as that described in the earlier work of Panzer and colleagues (2009) and revealed clear evidence that observation fostered the formation of a representation that used visual spatial coordinates that could support subsequent performance (see also Boutin et al. 2010). Only additional physical not observational practice *following observation* led to the emergence of a representation that used motor coordinates (Ellenbürger et al. 2012). It appears than that physical practice can foster the formation of both a visual-spatial (Boutin et al. 2010; Kovacs et al. 2009) and motor representation whereas observational practice is restricted to the development of a visual-spatial representation (see also Conessa et al. 2023).

A separate line of research has revealed that the aforementioned visual-spatial representation of skill that develops as a result of physical practice can benefit from a period of sleep that is commonly afforded either overnight or via a brief nap (Albouy et al. 2013; Cohen et al. 2005; King et al. 2017 for a review). In general, sleep has been reported to be a critical contributor to consolidation of a procedural skill (Robertson et al. 2004; Stickgold et al. 2001; Walker 2005). Consolidation is characterized as a form of off-line improvement or stabilization of performance resulting from the transformation of an initially fragile skill memory into a more stable form that is more resistant to the passage of time and interference (Robertson et al. 2004). At the neurophysiological level, it has been proposed that consolidation of skill memory is mediated by transient thalamocortical spindle activity and associated reactivations of task-related neural patterns during non-REM stage 2 sleep thought to occur following overnight sleep and during naps (Bottary et al. 2016; Boutin et al. 2018). More recently, the importance of temporal clustering of sleep spindles during this sleep stage has been identified as critically important for procedural skill consolidation (Boutin et al. 2018; Conessa et al. 2023).

Consolidation during sleep appears to target facilitation of the visual-spatial representation that develops during a prior period of physical practice. For example, Cohen et al. (2005) had participants implicitly learn a serial reaction time task then administered transfer tests that probed the availability of spatial and motor representations following practice. Transfer performance revealed that the spatial representation was enhanced following a night of sleep, whereas the motor representation was strengthened merely from a sufficient wake period. A similar outcome was reported more recently by Albouy et al. (2013) who introduced a 90-min daytime nap following practice as opposed to overnight sleep.

Like physical practice, gains in skill resulting from observation have been reported to be susceptible to further improvement from post-training consolidation although the specific mechanism through which these gains are accomplished may differ from those associated with physical practice (Van Der Werf et al. 2009; Maarvi-Hesseg et al. 2024). Consolidation that occurs after observational practice may also benefit from overnight sleep especially if it occurs close to training (Van Der Werf et al. 2009; Maarvi-Hesseg et al. 2024; Temporiti et al. 2023) although this claim remains controversial. Specifically, recent findings questioned the need for exposure to sleep to secure offline gains following observational training suggesting instead that the learner merely needs sufficient time to conduct requisite offline processing (Maarvi-Hesseg et al. 2024). While the specific skill memory that emerges from observation may garner limited benefit from exposure to sleep, there does appear to be evidence that generalizability of developed memory following observation can benefit from sleep. Recent work by Conessa et al. (2023) revealed a clear association between the availability of a visual-spatial representation following observation and the extent of REM2 sleep duration and temporal clustering of sleep spindles during a 90-min nap that followed training. These data are congruent with sleep’s contribution to the development of this representational form following physical practice (Cohen et al. 2005; Albouy et al. 2013). Unfortunately, as noted by Conessa et al. (2023) this study only included the relevant transfer test to assess visuo-spatial transfer but not its motor counterpart.

The present study was designed to address this issue by examining the impact of a 90-min nap following either physical or observational practice. Specifically, the present experiment examined if a daytime nap could enhance skill memory that was acquired through observation. Moreover, it was determined if the performance benefits that resulted from being afforded a nap was restricted to a specific representational form (visual-spatial or motor) that developed through observation. Based on the extent literature we assumed physical practice would support the development of both visual-spatial and motor representations and that only the visual-spatial form would benefit from exposure to a nap. Conversely, we anticipated that more limited performance gains would be achieved following observation when compared to physical practice and this would be accompanied by the emergence of a visual-spatial representation only. Should this occur, as predicted for physical practice participants, we expected the visual-spatial representation to be amenable to additional improvement from sleep manifest as enhanced non-mirror transfer performance for the observers that were administered a nap.

## Method

### Participants

Forty-eight undergraduate students participated in the experiment for course credit (24 male, 24 female; mean age = 21.46 years; SD ± 1.65 years). Determination of the necessary sample size was determined using G*power 3.1 (Faul et al. 2007). Using power of 95% and the *η*_*p*_*²* = 0.20 from the between-/within-subject interaction of the Ellenbürger et al. (2012) experiment part 1 it was determined that 36 participants was sufficient. Note that in the Ellenbürger et al. (2012) study, observational and physical practice scheduling was systematically varied, but consolidation was not investigated. Thus, for the present experiment, we chose to increase the calculated sample size to facilitate the possibility of discovering significant effects if they exist (see Lakens 2022).

No participant had prior experience with the experimental task or were informed of the specific purpose of the study. Handedness was determined by the Edinburg-Handedness Inventory (Oldfield 1971). Informed consent approved by the local ethics committee was obtained prior to participation by all participants. Sleep quality was assessed by the Pittsburgh Sleep Quality Index questionnaire (Buysse et al. 1989). The experiment was conducted in accordance with the revised version of the Declaration of Helsinki (World Medical Association 2013).

### Apparatus

The apparatus consisted of two horizontal levers affixed to one end of a near-frictionless vertical axle. The levers were fixed on the left and the right sides of a table, allowing the levers to move in the horizontal plane over the table surface. At the distal end of each lever, a vertical handle was fixed. The handle’s position could be adjusted so that when grasping the handle, the participants’ elbow could be aligned with the axis of rotation. A potentiometer (Midori Orange Pot CP-45 H; linearity +/- 0.4%) was attached to the lower end of the axis to record the position of the lever, and its output was sampled at 1000 Hz (Agilent Technologies, Agilent U2300 series USB Multifunctional Data Acquisition Device, USA).

**Fig. 1:**
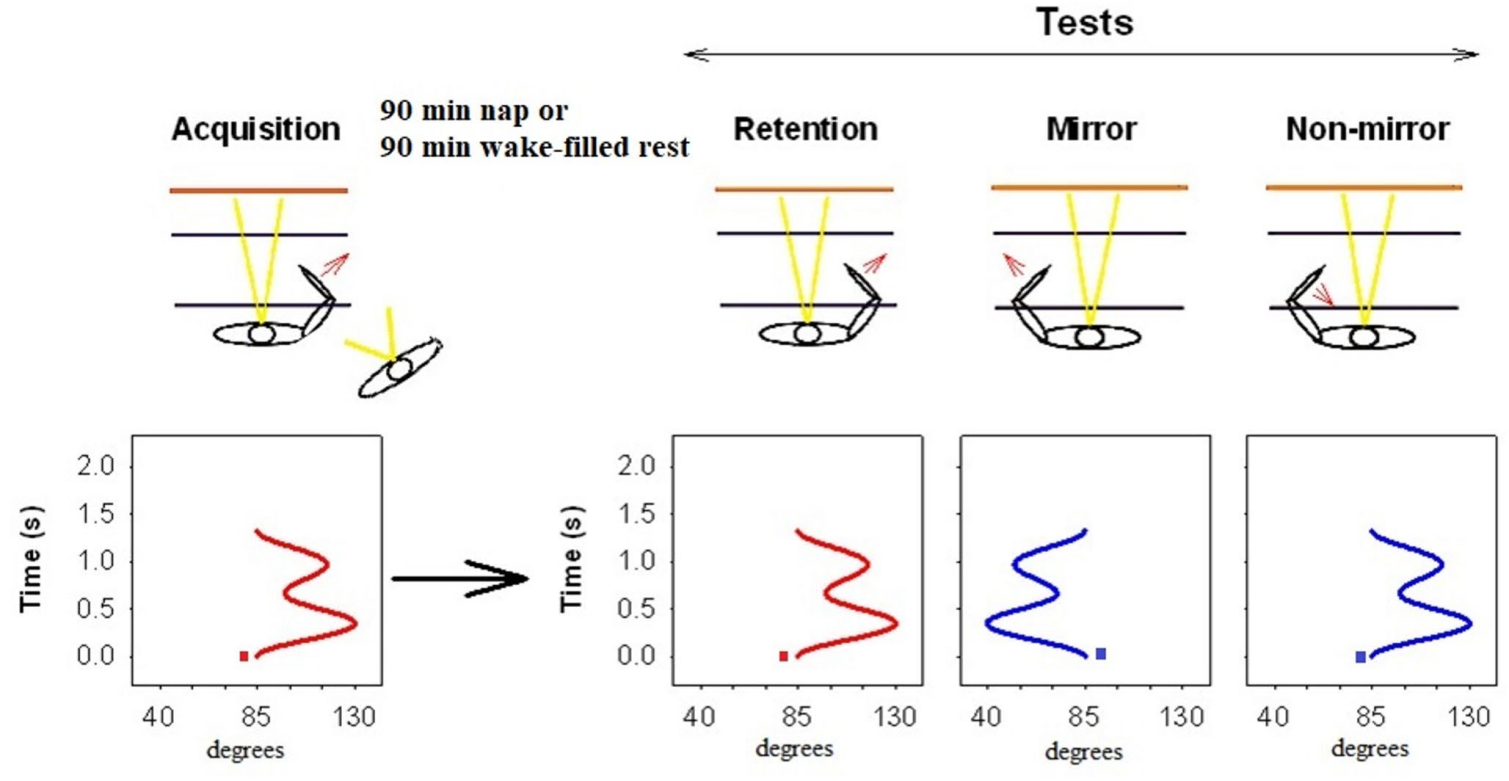
Illustration of limb and direction of the movement for the acquisition, retention, and mirror and non-mirror transfer tasks. The orientation of the target waveform for each acquisition and testing condition is also provided (bottom). The retention test was conducted following the acquisition session for 90 min. Following the retention test the two transfer tests (Mirror and Non-Mirror) were conducted in a counter balanced order

A projector (spatial resolution of 1024 × 768 pixels and a temporal resolution of 100 Hz), placed above and behind the participants, was used to display the goal waveform, the movement response (see Fig. 1) and feedback on the wall facing the participant. The participants sat in a frontal plane, orthogonally and about 2 m away from the wall. The 2 × 2 m image was projected in front of them on the wall.

### Task, procedure, and experimental groups

All experimental procedures occurred between 9 a.m. and 1 p.m. to control for circadian factors. Physical practice participants (P) were instructed to sit in front of the apparatus and their chair was adjusted so that the participant’s lower right arm was positioned at approximately an 85° angle to his/her upper arm in the starting position. At the beginning of each trial, the goal waveform was displayed (see Fig. 1), and participants were asked to move the lever to the starting position (1° x 1° area was displayed as the start position at the beginning of the goal waveform; see Fig. 1). The goal waveform was a spatial–temporal waveform pattern with three reversal points and a duration of 1300-ms. The goal waveform was created by summing two sine waves with different periods and amplitudes and was displayed as a static picture. The amplitudes in the goal waveform ranged from 0° to 45° from the start position. One second after positioning the cursor at the start position, a tone (900 Hz, duration 100-ms) was presented and informed the participant to begin the response when they were ready. As soon as the participant started the movement, the goal movement pattern disappeared from the wall and only the cursor representing the position of the lever was displayed.

Data collection was triggered by the movement of the lever. The participants were instructed to move the lever with their dominant right arm through a sequential pattern of extension–flexion movements (3 reversals; changing the movement direction from extension to flexion or vice versa). They were required to produce the criterion spatial-temporal pattern previously displayed on the screen, as accurately as possible and then return the lever to the start position. Approximately 1 s following the completion of the participants’ response, the criterion waveform and the movement pattern produced by the participant were superimposed as a static display for 5 s and the root mean square error (RMSE) was displayed as knowledge of results (KR). The time interval of 5 s was used to ensure that participants had enough time to process feedback information. During this time interval, participants were not allowed to move the lever from start position. The participants in the observation groups (O) were positioned to the side and back of the physical practice participant so that they had a clear view of the movement and display provided to the physical practice participant (see Fig. 1, left). Once the instructions began, participants (P and O) were not allowed to converse about the experiment. Observers were instructed to watch the model intently, to place their hands on their thighs to avoid arm movements and were informed that they would be asked to perform the task on subsequent tests.

Prior to entering the testing room participants were assigned to one of four experimental groups: physical practice with no nap (P-N), physical practice with nap (P + N), observation with no nap (O-N), and observation with a nap (O + N). Thus, all participants either observed (O) or physically (P) practiced during acquisition. Acquisition and testing were conducted in a silent and dimly lit room. Upon arrival at the testing room, each participant received verbal and written instructions regarding the goals of the task. During acquisition participants in the P groups performed the flexion–extension movement with their dominant right arm. Participants in the O groups were randomly yoked to participants in the physical practice condition. Thus, each participant in the O group observed a single participant from the P group performing the spatial temporal movement pattern during acquisition. This form of observation is often referred to as a learning model (see Shea et al. 2011).

Acquisition consisted of 99 trials divided into 11 blocks of 9 trials each. Between each block was a rest interval of 30 s. Following the completion of acquisition (approximately one hour) individuals were pseudo-randomly divided. Note, a pseudo randomization was performed to have equal distribution of nap (+ N) and (-N) no nap individuals in each group. One participant was instructed to take a nap (+ N) of 90 min, while the other has an equivalent period of wake-filled (-N) rest. Individuals in the + N groups were instructed to lie down on a comfortable bed with their own pillow and their own blanket in a quiet and dark room conducive to encouraging sleep. Individuals assigned to the -N groups were positioned in a quiet, well-lit room where they were allowed to read sports-related magazines or comics. After 90 min, all nap participants were awakened and then like the non-nap participants were tested individually on the retention and two inter-manual transfer tests.

For the retention test, the same limb was used as during acquisition and both the motor and visual–spatial coordinates used during acquisition were reinstated. The two inter-manual transfer tests (ten trials each) followed with the order of presentation of the transfer tests being counterbalanced. The two transfer tests (mirror and non-mirror) were performed with the contralateral limb (see Fig. 1). In the mirror transfer test, the goal waveform was mirrored on a vertical axis so that the same pattern of homologous muscle activation and limb joint angles were required as experienced during acquisition and on the retention test. For the non-mirror transfer test, the visual–spatial location of the target waveform practiced or observed during acquisition and experienced during retention testing were reinstated, but the participant used the contralateral limb. Note that in the mirror transfer test, the motor coordinates used or observed during acquisition were reinstated while the visual–spatial coordinates were changed. Alternatively, on the non-mirror transfer test, the visual–spatial coordinates used or observed during acquisition were reinstated but the motor coordinates were changed. Following completion of the transfer tests, the Pittsburgh Sleep Quality Index (PSQI) questionnaire was completed by all participants.

### Data analysis

Data analysis was performed using MATLAB (Mathworks, Natick, MA; Version 2024a). The individual trial time series were used to compute lever displacement. To reduce noise, the displacement time series was filtered with a 2nd order dual-pass Butterworth filter with a cutoff frequency of 10 Hz. The RMSE in degrees (°) was computed to estimate the performance error in achieving the goal movement pattern. RMSE captures errors in both amplitude and time. Additionally, RMSE incorporates both the variability and the bias of the performed movement pattern. RMSE was calculated by the difference between the goal wave form and the actual movement pattern produced by the participant at each data point in the time series. Then, the differences for each data point in the time series were squared, and the mean of the squared differences was computed on a trial basis. The final step in computing RMSE was to determine the square root of the mean of the squared differences. The mean RMSE was computed per participant for each of eleven blocks each containing 9 trials. Note, for the retention and the transfer tests the first trial was removed, because of the set-up to calibrate the motor system (Nacson and Schmidt 1971). The PSQI was used to evaluate prior general sleep duration and quality but this did not address the nap specifically which was merely noted by having to wake individuals to participate in the test phase once the nap interval (i.e., 90-min) was over. The statistical analysis was conducted using SPSS 29.0 software (IBM Corp., Armonk, NY, USA).

Mean RMSE for acquisition for the individuals that experienced physical practice was analyzed using a 2(Nap: +N, -N) x 2(Block: 1–11) analysis of variance (ANOVA) with repeated measures on Block. For physical practice participants only, the change in performance from the end of acquisition, determined as mean RMSE for Blocks 9–11, was analyzed by a 2(Nap: +N, -N) x 2(Test: Acquisition, Retention) ANOVA with repeated measures on Test.

Mean RMSE for the retention test for observers and physical practice participants in both nap and no-nap conditions was analyzed in a 2(Practice Modality: P, O) x 2(Nap: +N, -N) ANOVA. Mean RMSE for the same individuals for the transfer tests was analyzed in a 2(Practice Modality: P, O) x 2(Nap: +N, -N) x 2(Transfer: mirror; non-mirror) ANOVA with repeated measures on the Transfer factor. To evaluate if observation per se enhanced acquisition of the novel motor sequence, mean RMSE for observational practice participants during the retention trials was compared to the RMSE for the initial training block for the physical practice participants using an independent t-test. Overall sleep duration from prior to arriving at the experiment was analyzed in a 4 (P + N, P-N, O + N, O-N) ANOVA, whereas sleep quality across the groups was analyzed by the H-test by Kruskal-Wallis. Finally, the duration of the nap period (in min) from the time the room was darkened and the participant lay on the bed to the moment the participant was awakened to participate in the test period was recorded. Mean duration for the participants that napped that were assigned to physical and observation were separately determined and assessed using a timer controlled by the experimenter. He noted the time when he left the room and the time when he entered the room. Adjustments were made as needed for violations of homogeneity and sphericity (Winer 1971). Partial eta square (η_p_²) is the effect size for the ANOVAs and is reported for all significant effects (Cohen 1988). For t-tests, Cohens’ d is reported. The significance level was set at *p* < .05.

## Results

**Fig. 2:**
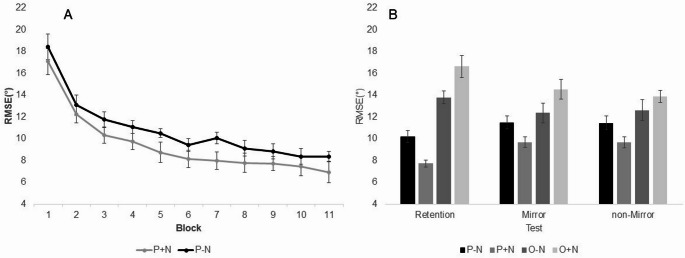
Mean RMSE and the SEM in ° during acquisition (A) and retention/transfer test (B)

The mean RMSE and the standard error of the means (SEM) for the acquisition phase of the P + N and the P-N groups are displayed in Fig. 2A. Subsequent performance during retention and transfer test are also included for all experimental conditions (P-N, P + N, O-N, O + N) and displayed in Fig. 2B.

### Acquisition phase for physical practice participants

The analysis of mean RMSE indicated a main effect of Block, *F*(10, 220) = 55.49, *p* < .01, *η*_*p*_*²* = 0.72. Duncan’s new multiple range test found that mean RMSE decreased through block 7 while mean RMSE across blocks 8–11 did not differ. The Nap x Block interaction, *F*(10, 220) = 0.19, *p* > .05, and the main effect of Nap, *F*(1, 22) = 2.56, *p* > .05, failed to reach significance.

### Change in performance from the end of acquisition to retention for the physical practice groups

Mean RMSE did not change from the completion of the training phase to the retention test trials as indicated by the lack of a significant NAP x Test interaction, *F*(1, 22) = 0.459, *p* > .05, as well main effects of Test, *F*(1, 22) = 3.867, *p* > .05, and NAP, *F*(1, 22) = 0.794, *p* > .05.

### Retention test performance

The analysis of RMSE revealed a significant Practice Modality x Nap interaction, *F*(1, 44) = 14.533, *p* < .01, *η*_*p*_*²* = 0.25 and a main effect of Practice Modality, *F*(1, 44) = 76.50, *p* < .01, *η*_*p*_*²* = 0.64. The main effect of NAP was not significant, *F*(1, 44) = 0.694, *p* > .05. Simple main effect analysis for Practice Modality as a function of NAP indicated that mean RMSE differed for P, *F*(1, 44) = 5.83, *p* < .05, *η*_*p*_*²* = 0.12 and for the O groups *F*(1, 44) = 8.86, *p* < .01, *η*_*p*_*²* = 0.17, across NAP. Subsequent multiple range test for the P groups revealed that mean RMSE was higher for the -N condition (*M* = 10.19°, *SEM* = 0.58°) compared to the + N condition (*M* = 7.71°, *SEM* = 0.33°). For the O groups, this pattern was reversed with mean RMSE lower for the -N condition (*M* = 13.77°, *SEM* = 0.77°) compared to the + N condition (*M* = 16.82°, *SEM* = 1.02°).

However, observation did appear to facilitate performance as mean RMSE was significantly lower during the retention trials for the observation conditions (*M* = 15.46°, *SEM* = 0.71°) when compared to mean RMSE for the physical practice participants for Block 1 of acquisition (*M* = 17.74°, *SEM* = 0.83°), *t*(46) = 2.24, *p* = .03, *d* = 0.64.

### Transfer test performance

The analysis of RMSE during the transfer tests revealed a significant Practice Modality x Nap interaction, *F*(1, 44) = 9.00, *p* < .01, *η*_*p*_*²* = 0.17 and a main effect of Practice Modality, *F*(1, 44) = 22.55, *p* < .01, *η*_*p*_*²* = 0.34. Simple main effect analysis of the significant interaction for practice modality across nap indicated that the RMSE differed for the P groups between + N and -N, *F*(1, 44) = 4.69, *p* < .05, *η*_*p*_*²* = 0.10. The same outcome emerged for the O groups *F*(1, 44) = 4.31, *p* < .05, *η*_*p*_*²* = 0.09. The RMSE for the P groups was higher for the -N condition (*M* = 11.46°, *SEM* = 0.69°) compared to the + N condition (*M* = 9.67°, *SEM* = 0.49°). In contrast, for the O groups, mean RMSE was lower for the -N condition (*M* = 12.48°, *SEM* = 0.92°) compared to the + N condition (*M* = 14.20°, *SEM* = 0.73°). Simple main effect analysis for nap across practice modality indicated that the RMSE differed for + N between the P and the O groups, *F*(1, 44) = 30.02, *p* < .001, *η*_*p*_*²* = 0.41, but not for the -N condition, *F*(1, 44) = 1.53, *p* > .05. The three-way interaction Practice Modality x Nap x Test, *F*(1, 44) = 1.38, *p* > .05, and the two-way interactions Nap x Test, *F*(1, 44) = 1.07, *p* > .05, and Practice Modality x Test, *F*(1, 44) < 0.01, *p* > .05, failed to reach significance. The main effects of Nap, *F*(1, 44) = 0.07, *p* > .05, and Test, *F*(1, 44) = 0.07, *p* > .05, also revealed no significance.

### Sleep duration and sleep quality prior to experiment

The average sleep duration for the P + N group was 8 h 49 mi ± 1 h 12 min, for the P-N group 7 h 55 mi ± 54 min, for the O + N group 8 h 02 min ± 1 h 10 min and for the O-N group 8 h 17 min ± 1 h 37 min. The analysis failed to indicate a significant difference between the four groups, *F*(3, 44) = 1.26, *p* > .05. Further, the analysis of the sleep quality indicated no significant effects across the four groups, *H*(3) = 0.401, *p* > .05.

### Sleep duration for physical and observation participants assigned to nap

The duration of the nap period for the physical (P + N: *M* = 90.33 min, *SEM* = 0.35) and observational practice participants (O + N: *M* = 90.16 min, *SEM* = 0.47) that were assigned to nap conditions did not differ, *t*(22) = 0.28, *p* = .78.

## Discussion

The primary purpose of the present experiment was to determine if a 90-min daytime nap can enhance the development of sequence representation developed via observation for a novel dynamic movement sequencing skill. It was assumed that the representation of skill that developed through observational practice would most likely be visual-spatial in nature (see Boutin et al. 2010; Ellenbürger et al. 2012; Gruetzmacher et al. 2011; Conessa et al. 2023) and exposure to a period of sleep might strengthen the memory representation that emerged following observation (Cohen et al. 2005; Albouy et al. 2013). Thus, for the present experiment, we reasoned that affording a nap after observation of a novel movement sequence should facilitate the quality of the visual-spatial representation that emerged via observation when compared to individuals that merely observed. With respect to the construction of a motor representation, only sufficient physical but not observational practice affords the development of motor related representation (Kovacs et al. 2009; Panzer et al. 2009). In the present study then it was reasoned that only physical but not observational practice would support the development of this representational form.

A number of key findings emerged in this experiment, First, as expected performance during retention and transfer were superior for physical practice compared to merely observing (Deakin and Proteau 2000; Boutin et al. 2010). Second, mean RMSE for observers during the retention trials was lower than that displayed by physical practice participants during Block 1 of training. These data support the claim that useful information is available via observation supporting skill acquisition. Third, a short nap following practice only benefited individuals that physically practice but for this practice modality this benefit was quite broad in nature resulting in transfer across both visual-spatial and motor frames of reference. In contrast, a nap following observation, not only failed to support the emergence of visual-spatial representation actually deterred the development. Each of these specific findings are discussed in more detail in the following sections.

### Acquisition of a novel sequential skill through physical and observational practice

As expected, physical practice led to skill acquisition during the initial practice phase revealed in the significant decrease in mean RMSE especially during the first seven blocks of training. Not surprisingly there was no difference in RMSE for the physical practice participants that would subsequently sleep (i.e., P + N) or remain awake (i.e., P-N) during the interval between training and test. Importantly, observation offered benefits for novel skill acquisition revealed by the lower RMSE for observers at the time of retention compared to the RMSE displayed during the initial block by individuals assigned to physical practice. These data are consistent with a growing body of literature revealing the efficacy of observation for movement sequence learning (e.g. Boutin et al. 2010; Ellenbürger et al. 2012; Maaravi-Hesseg et al. 2016; Conessa et al. 2023). In general, results from the retention and transfer tests highlight the well documented advantage of physical practice over observational practice for learning motor skills (e.g. McCullagh et al. 1989; Shea et al. 2000; Boutin et al. 2010). However, the present work also revealed some unique influences of day-time sleep for the effectiveness of physical and observational practice as well as on the development of a sequence representation in the various training modalities. These issues are addressed in the next sections.

### Retention and transfer of sequence knowledge: physical practice and daytime sleep

Individuals who physically practiced the task regardless of whether they assigned to the nap group or the or no nap group, showed no performance decrements from the end of the acquisition to the retention test. Specifically, retention performance was superior for individuals that were privy to a brief nap compared to those that remained awake prior to the retention test. It has been previously argued that improved retention in this case stems from a more successful implementation of consolidation whilst asleep during which a series of critical neurophysiological events are reported to be enhanced. The present work was not designed to delineate how sleep provides the benefit revealed here or in numerous other previous studies. However, recent theorizing has focused on the importance of temporal clustering of sleep spindles during NREM2 sleep as central to the reported procedural memory benefit from sleep (Boutin and Doyon 2020).

For the individuals that physically practiced, the benefit from a post-practice nap was quite broad as superior transfer was evident for both the non-mirror and mirror transfer tests. Thus, novel sequence representations that have been previously demonstrated to emerge from physical practice relying on either allocentric or egocentric codes were both susceptible to improvement from processing that was associated with a brief daytime sleep exposure. It should be recognized that sleep-dependent enhancement for transfer was only expected to occur during the non-mirror test, that is, when the visual-spatial representation was primarily reinstated to supervise sequence execution (Cohen et al. 2005; Albouy et al. 2013). While we hypothesized that the development of a motor representation might occur from physical practice, sufficient to support performance in the mirror transfer test administered in this experiment (see Panzer et al. 2009), there was no expectation that this memory form would be susceptible to sleep-dependent consolidation.

This finding is counter to that reported by Albouy and colleagues (2013) who revealed no advantage to being privy to a nap prior to evaluation of the status of the motor representation. An important difference in the work of Albouy et al. (2013) and the present work, however, was the inclusion of a *representation test* immediately after practice was concluded and before sleep in the former study. In essence, the participants physically practiced a sequence that required the use of the spatial or motor representation that was available to the learner at the end of training. Indeed, this practice was not insignificant, amounting to approximately one-third of the initial training. In the present work, the emergent representations, visual-spatial or motor, were only retrieved after the nap in order to support performance on the relevant transfer test. Thus, in the work of Albouy et al. (2013), one cannot discount the possible contribution from using each skill representation for sequence execution when evaluating the influence of the subsequent nap on status of the visual-spatial and/or motor representation. In summary, while the present data offers further support for a role of sleep-dependent consolidation for fostering skill representation framed in allocentric space (see King et al. 2017), these data also raise the possibility that other skill representation that evolve from physical practice might be amenable to being enriched by sleep-related offline activity. Clearly, the reliability of this latter finding requires verification in subsequent work.

### Retention and transfer of sequence knowledge: observation and daytime sleep

A key objective of the present experiment was to determine if the beneficial influence of a nap following physical practice also occurs after observational practice (Van Der Werf et al. 2009; Conessa et al. 2023). Moreover, the influence of a nap for the development of visual-spatial and motor representations via observation was determined (see Conessa et al. 2023). It was predicted, based on findings from previous sequence learning experiments (Boutin et al. 2010; Gruetzmacher et al. 2011) and other memory consolidation research (Albouy et al. 2013; Connessa et al. 2023) that observation of a movement sequence would support the development of a visual-spatial representation. Furthermore, it was assumed that this representational form should benefit from exposure to a nap in a manner akin to that exhibited after physical practice. Indeed, it has been reported that sleep spindle architecture shown to support procedural learning has been revealed following observation for individuals that have demonstrated successful transfer to a visual-spatial version of the observed sequence skill (Boutin et al. 2018; Conessa et al. 2023).

Contrary to expectations, participants that observed and were privy to a nap immediately after practice performed the retention and the two transfer tests with higher RMSE compared to those observers who had an equivalent wake-filled rest period. Thus, sleep in the form of a brief 90-min nap following observation hindered offline gain which extends to a degradation in visual-spatial and motor representations for the observers that slept. This result is contradictory to our initial hypothesis that a nap favors the consolidation of a visual-spatial representation irrespective of whether it is a result of physical or observational practice. At first glance, these data appear incongruent with earlier efforts by Gruetzmacher et al. (2011) that clearly revealed successful generalization of observational learning in allocentric space. That is, participants who were only privy to observation exhibited superior non-mirror test transfer. In contrast to the present experiment, it is important to note that the sleep experienced by individuals in the Gruetzmacher et al. (2011) study tool place overnight and presumably of greater duration than the 90-min included in this experiment (see also Ellenbürger et al. 2012). Thus, two potential issues may be critical for establishing improvements in the visual-spatial reference developed via observation. First, the benefit might indeed be sleep-directed but sleep must occur overnight, as was the case in the Gruetzmacher et al. (2011) study rather than just a brief nap as was the case in the present work. This proposal is congruent with the claim that the nature of consolidation during naps and period of diurnal sleep are mechanistically distinct (see, van Schalkwijk et al. 2017). The plausibility of this hypothesis however is reduced by the fact that Conessa et al. (2023) reported successfully visuo-motor transfer following observation that was supplemented with a similar nap to that used here. Second, it is possible that far greater time must occur between training and test to afford adequate consolidation to occur following observation. If this were the case, then clearly any benefits are not linked to sleep per se, rather they are time-dependent which has been previously reported to be sufficient for some forms of consolidation (Robertson 2009). Indeed, this is not a new proposal for performance enhancement through observation. Recent findings by Maaravi et al., (2024) have questioned the need for sleep after observation to garner offline gain. Rather, Maaravi and colleagues argue that it is a time-dependent form of consolidation more akin to that reported during declarative or perceptual learning that is more critical in this situation.

One final unanticipated but interesting outcome associated with the observation condition is the relatively superior performance in the transfer as opposed to the retention test. At first glance one might assume that retention, reproduction of what was observed should be superior to the generalization to related behaviors. However, there is some evidence consistent with the notion that the observational practice context is well suited for extracting the relative or more abstract features as opposed to the absolute features of an action. According to Scully and Newell (1985) the relative features of an action have more saliency for the success of observational practice than the absolute motion features of an action. Consistent in with this position, Buchanan et al., (2007) had learn a rhythmic single limb multi-joint coordination task through physical or observational practice. The task consisted of a 90° relative phase pattern between the elbow and wrist in combination while also requiring very specific excursion amplitude for the wrist and elbow. After 2-days of observational practice participants could closely match the performance of the required relative phase. However, observation offered no benefit beyond a no-practice control condition with respect to meeting the required joint amplitudes. The authors argued that, in agreement, with Scully and Newell (1985), that relative features of the complex coordinated behavior were amenable to observation whereas the more task-specific joint amplitudes were only incorporated through physical practice to achieve the appropriate scaling. The present case may be further evidence for the proprietary access observation has to the more abstract characteristics of novel actions.

## Conclusion and limitations

The results of the present experiment revealed the repeatedly documented influence of sleep-dependent offline gain of motor sequence knowledge following physical practice (Doyon et al. 2018; King et al. 2017 for a review). In this case, sleep was introduced without a post task time delay in the form of a 90-min daytime nap (see also Conessa et al. 2023). This sleep benefit extends to the enhancement of both visual-spatial and motor representations that are developed across the course of physical practice. In contrast, both retention and transfer performance is significantly lowered if observational practice is followed by a similar nap. The finding that the visual-spatial representation that is constructed during observation did not benefit from sleep was inconsistent with earlier reports. Two possible reasons for this discrepancy are considered including the possibility that following observation, any consolidation is time-dependent or that it is diurnal sleep that is central to any latent improvement via consolidation. While the present experiment was not designed to delineate specific features of sleep architecture that account for benefits of observation to skill acquisition, the absence of sleep monitoring is a limitation of the present experiment and going forward, future studies addressing similar issues to those contained herein would benefit from their addition.

## Data Availability

No datasets were generated or analysed during the current study.
